# Antibiotic Susceptibility Profiling of Gram-Positive and Gram-Negative Bacterial Isolates in a Tertiary Care Hospital: Establishment of an Antibiogram

**DOI:** 10.7759/cureus.60542

**Published:** 2024-05-18

**Authors:** Kannan R, Ashik Anil, Pritty Thomas, Nijin Samuel Raju, Sherin M Reji

**Affiliations:** 1 Pharmacology and Therapeutics, East Point College of Pharmacy, Bangalore, IND; 2 Pharmacology and Therapeutics, East Point Hospital and Research Center, Bangalore, IND; 3 Clinical Pharmacy, St John's Medical College Hospital, Bangalore, IND; 4 Pharmacology and Therapeutics, Believers Church Medical College Hospital, Bangalore, IND; 5 Pharmacy, Hillside College of Pharmacy and Research Centre, Bangalore, IND

**Keywords:** gram negative bacteria, gram positive bacteria, clsi guidelines, whonet, antimicrobial resistance, antibiogram

## Abstract

Introduction

Antimicrobial resistance poses a significant global healthcare challenge in the management of bacterial infections, which is frequently attributed to rapid bacterial adaptations. This study aims to develop an antibiogram for a tertiary care hospital, providing comprehensive antibiotic sensitivity profiles for Gram-positive and Gram-negative bacteria. It informs healthcare providers of antibiotic resistance trends, enabling informed treatment decisions and enhanced infection control measures.

Methods

We conducted a six-month prospective observational study, during which we gathered and analyzed data from the microbiology laboratory to identify patterns of antimicrobial sensitivity. Subsequently, the data underwent analysis and interpretation using the respected WHONET software, a readily available tool designed for this specific task. Our methodology adhered to the guidelines established by the Clinical & Laboratory Standards Institute for the standardization of antibiogram generation procedures, and these guidelines are easily integrated into the WHONET software for analytical purposes.

Results

There were a total of 357 isolates across various hospital departments, comprising 13 distinct bacterial species. Among them, nine were identified as Gram-negative bacteria, accounting for 262 (73.3%) isolates. *Escherichia coli* accounted for 131 (36.6%) isolates, while *Klebsiella* accounted for 62 (17.3%), emerging as the predominant species among them. The remaining four bacterial species were identified as Gram-positive bacteria, totaling 95 (26.6%) isolates, with *Staphylococcus aureus* being the most frequently isolated species at 51 (14.2%), followed by *Enterococcus* at 26 (7.2%). Subsequent analysis using the WHONET software facilitated the creation of an antibiogram. Among the Gram-negative bacteria, *E. coli* displayed high sensitivity (100%) to aztreonam and clindamycin, followed by nitrofurantoin (98%), imipenem (94%), and meropenem (95%). However, it exhibited decreased sensitivity to ampicillin (25%), cefuroxime (34%), and ceftriaxone (39%). Conversely, among the Gram-positive bacteria, *S. aureus* demonstrated 100% sensitivity to ampicillin, amoxiclav, cefazolin, teicoplanin, linezolid, rifampicin, nitrofurantoin, and cefotaxime. However, it exhibited zero sensitivity to vancomycin and only 6% sensitivity to cotrimoxazole.

Conclusion

This study advances the understanding of antibiotic susceptibility in a tertiary care setting and provides an invaluable tool for optimizing treatment strategies, enhancing infection control measures, and combating antibiotic resistance.

## Introduction

The discovery of antibiotics in the mid-20th century has opened up a new era in medicine. These remarkable drugs achieved considerable success in combating bacterial infections, leading to the saving of numerous lives [1]. However, various challenges and consequences have been raised in treating bacterial infections as, over the decades, the bacteria and resourceful microorganisms have adapted and evolved mechanisms of resistance to these life-saving drugs. This phenomenon of antibiotic resistance constitutes a significant threat to global public health, demanding heightened vigilance, innovative strategies, and a profound comprehension of bacterial resistance mechanisms. Antibiotic resistance is not a new phenomenon but has been accelerated due to various factors. The chief among them is the misuse of antibiotics in humans, veterinary medicine, and agriculture [2]. This pressure exerted by the overuse of antimicrobial agents has led to the development of resistance mechanisms, survival, and proliferation in the microorganisms, making life-saving drugs powerless against them [3]. The consequences of antibiotic resistance have increased mortality and morbidity, leading to longer hospital stays and the need for more toxic and expensive alternative therapies [4]. While antibiotic resistance is a worldwide problem, the mechanism of resistance may vary within geographies and healthcare systems [5]. Tertiary care hospitals cater to patients with severe conditions, often involving complex surgeries, immunosuppressive therapies, and extended hospital stays. These settings are particularly susceptible to the emergence and dissemination of antibiotic-resistant bacteria [6]. *Staphylococcus aureus* is a major contributor to hospital-acquired infections globally, primarily associated with skin and bloodstream infections [7]. Among Gram-negative bacteria,* Pseudomonas* and *Acinetobacter *are significant contributors to hospital-acquired infections. The increasing resistance of these bacteria to beta-lactam antibiotics is emerging as a major concern in healthcare settings [8]. Hence, it is essential for tertiary care facilities to implement robust systems for monitoring antibiotic usage and resistance development.

An antibiogram is a comprehensive tool that provides clinicians with a summary of bacterial susceptibility to several antibiotics. They give useful information regarding which antibiotics are most likely to be effective against clinically encountered bacterial strains [9]. The importance of antibiograms is shown by their role in influencing the selection of empirical antibiotic treatment. When patients come in with infections, doctors may have limited knowledge about the organism causing the infection and its susceptibility profile. In such circumstances, empirical therapy based on a broad-spectrum antibiotic that is expected to cover a wide variety of possible infections is commenced. However, the most appropriate empirical therapy is selected by the local epidemiology of bacterial resistance, which is precisely what an institution’s own antibiogram provides [10]. Antibiograms have applications beyond individual patient treatment. They are critical components of antibiotic stewardship programs, which attempt to optimize antibiotic usage in order to enhance patient outcomes, prevent the spread of antibiotic resistance, and reduce adverse events associated with antibiotic use. Our study focuses on constructing an antibiogram by analyzing data from the hospital’s microbiology laboratory using the WHONET software, following the Clinical & Laboratory Standards Institute (CLSI) guidelines. This research aims to enhance the understanding of antibiotic efficacy and bacterial responses, facilitating the creation of a tailored antibiotic guide for improved antibiotic stewardship and combating antibiotic resistance.

## Materials and methods

A prospective observational study was conducted over six months, from January 2022 to June 2022, at East Point Hospital and Research Center in Bangalore, India. The study aimed to systematically assess the antimicrobial sensitivity patterns of bacterial isolates obtained from various clinical specimens.

Sample collection, identification, and sensitivity testing

A total of 345 inpatient samples, comprising urine, blood, sputum, swab, and pus samples, were collected, with each sample labeled with personal details and collection date. Upon collection, samples were carefully transported to the laboratory under appropriate conditions to maintain integrity. Different agar plates, namely cystine-lactose-electrolyte-deficient agar, blood agar, chocolate agar, and MacConkey agar, were employed for bacterial culture, tailored to the sample type. Incubation was conducted for 24 hours at 37 °C under optimal conditions. Following colony formation, Gram staining was employed for initial bacterial classification into Gram-positive or Gram-negative groups. Subsequently, catalase, coagulase, and biochemical tests were executed to identify specific bacteria, adhering rigorously to standard microbiological protocols. Sensitivity testing ensued using Mueller-Hinton agar plates, in line with recommendations from the CLSI. Antibiotic discs were applied to the agar plates via the disk diffusion method. Post-incubation for 24 hours, the inhibition zone surrounding each disc was measured utilizing the antibiotic zone scale, with interpretations made according to CLSI guidelines to determine antimicrobial sensitivity and resistance.

Data collection and analysis

The data collection process commenced with the retrieval of antimicrobial sensitivity test reports from the microbiology department of East Point Hospital and Research Center. These reports contained comprehensive information on the susceptibility of bacterial isolates to a range of antibiotics. All collected data was diligently managed and stored securely and confidentially. The data were then subjected to comprehensive analysis using the WHONET 2021 version software, a widely recognized tool for the analysis of antimicrobial susceptibility data. The WHONET software provided essential features for the interpretation of data, including the determination of resistance patterns, the calculation of minimum inhibitory concentrations, and the generation of summary reports.

Inclusion Criteria

Samples included in this study were sourced from hospitalized patients within the specified study timeframe. The inclusion criteria encompassed bacterial isolates of both Gram-positive and Gram-negative types, identified through standard laboratory methodologies. These bacterial specimens underwent antimicrobial susceptibility testing as per the routine clinical protocol.

Exclusion Criteria

Our analysis excluded isolates sourced externally to the hospital or those collected outside the designated study period. Additionally, bacterial isolates lacking complete or available antimicrobial susceptibility testing data were also excluded from the study. These criteria were implemented to ensure the consistency and reliability of the data analyzed in our study.

Ethical considerations

This research adhered to all relevant ethical principles and guidelines for the use of patient data, ensuring that consent forms were obtained from all participating patients. The study was conducted in accordance with the Declaration of Helsinki, and all data were anonymized to protect patient confidentiality and privacy. Ethical approval for the study was obtained from the Institutional Ethics Committee at East Point Hospital and Research Center.

## Results

**Figure 1:**
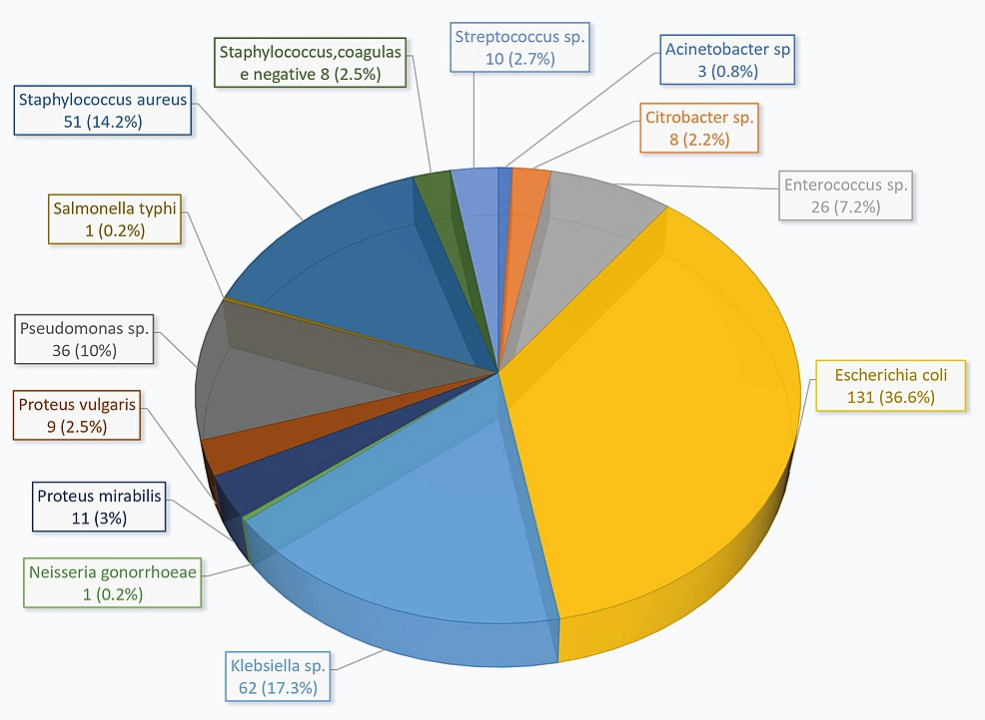
Thirteen bacterial species and the number of isolates identified in the study

A total of 357 isolates were identified from 345 patient samples obtained across multiple departments within the hospital over a period of six months. Samples were collected mainly from blood, pus, urine, sputum, swab, and vaginal aspirate. Out of all these, the majority of isolates were found in urine specimens, totaling 144 (41.7%). These isolates encompassed a diverse array of bacterial strains, comprising 13 distinct species of both Gram-positive and Gram-negative organisms, as outlined in Figure 1. Among the isolates, 95 were characterized as Gram-positive bacteria, while 262 were classified as Gram-negative bacterial strains. A comprehensive depiction of the total participant count along with their respective gender distribution within the context of this research study is provided in Table 1.

**Table 1:** A comprehensive overview of the total number of participants and their gender distribution in the context of this research

Organism	Number of isolates, n (%)	Number of patients, n (%)	Female	Male
*Acinetobacter *sp.	3 (0.8%)	3 (0.8%)	2	1
*Citrobacter* sp.	8 (2.2%)	8 (2.3%)	2	6
*Enterococcus *sp.	26 (7.2%)	26 (7.5%)	22	4
Escherichia coli	131 (36.6%)	128 (37.1%)	99	29
*Klebsiella* sp.	62 (17.3%)	59 (17.1%)	35	24
Neisseria gonorrhoeae	1 (0.2%)	1 (0.2%)	0	1
Proteus mirabilis	11 (3%)	11 (3.1%)	4	7
Proteus vulgaris	9 (2.5%)	8 (2.3%)	3	5
*Pseudomonas* sp.	36 (10%)	33 (9.5%)	15	18
*Salmonella *Typhi	1 (0.2%)	1 (0.2%)	1	0
Staphylococcus aureus	51 (14.2%)	49 (14.2%)	21	28
*Streptococcus* sp.	10 (2.7%)	10 (2.9%)	4	6
*Staphylococcus* coagulase-negative	8 (2.5%)	8 (2.3%)	6	2
Total	357 (100%)	345 (100%)	214	131

Antimicrobial sensitivity pattern of Gram-positive bacteria

Among the 13 identified species, four were classified as Gram-positive bacteria, specifically *Enterococcus *species, *S. aureus*, *Staphylococcus *coagulase-negative, and *Streptococcus *species. Notably, *S. aureus *was the most frequently isolated bacterium, comprising 51 bacterial isolates, followed by *Enterococcus *species with 26 isolates, *Streptococcus* species with 10 isolates, and *Staphylococcus* coagulase-negative with eight isolates. The antimicrobial sensitivity profiles of these Gram-positive bacteria to various antimicrobial agents are depicted in Table 2, as elucidated in this research article.

**Table 2:** Antimicrobial sensitivity profile of Gram-positive bacteria NT, not tested

Organism	Number of isolates, n (%)	Antibiotic sensitivity percentage (%)																	
		Ampicillin	Amoxiclav	Cefazolin	Gentamycin	Tetracycline	Ciprofloxacin	Cefotaxime	Erythromycin	Clindamycin	Cefoxitin	Cotrimoxazole	Chloramphenicol	Levofloxacin	Nitrofurantoin	Vancomycin	Teicoplanin	Linezolid	Rifampicin
*Enterococcus *sp.	26 (7.2%)	81	NT	NT	33	40	40	NT	50	0	NT	40	100	0	75	82	40	92	NT
Staphylococcus aureus	51 (14.2%)	100	100	100	83	90	38	100	43	74	41	6	96	31	100	0	100	100	100
*Staphylococcus* coagulase-negative	8 (2.5%)	100	NT	NT	100	67	57	NT	14	43	33	63	100	100	100	0	0	100	67
*Streptococcus* sp.	10 (2.7%)	100	0	100	50	33	0	100	33	67	0	0	100	57	NT	100	67	100	NT

Antimicrobial sensitivity pattern of Gram-negative bacteria

Among the 357 bacterial isolates analyzed, 262 were identified as Gram-negative bacteria, encompassing the following genera: *Acinetobacter*, *Citrobacter*, *Escherichia coli*, *Klebsiella*, *Proteus mirabilis*, *Pseudomonas*, *Proteus vulgaris*, *Salmonella *Typhi, and *Neisseria gonorrhoeae. *Notably, *E. coli *and *Klebsiella *species were the predominant Gram-negative isolates, comprising 131 and 62 specimens, respectively. *E. coli *was the dominant isolate among the urine specimens, with 86 (66%) isolates sourced from this sample type, particularly prevalent among females. The antimicrobial sensitivity profiles of these Gram-negative bacteria to a range of antimicrobial agents are demonstrated in Table 3.

**Table 3:** Antimicrobial sensitivity profile of Gram-negative bacteria NT, not tested

Organism	Number of isolates, n (%)	Antibiotic sensitivity percentage (%)														
		Ampicillin	Piperacillin/tazobactam	Aztreonam	Imipenem	Meropenem	Gentamycin	Amikacin	Ciprofloxacin	Ceftriaxone	Cefuroxime	Clindamycin	Cotrimoxazole	Levofloxacin	Nitrofurantoin	Linezolid
*Acinetobacter* sp.	3 (0.8%)	0	0	NT	0	0	0	0	0	0	0	NT	0	0	NT	NT
*Citrobacter* sp.	8 (2.2%)	25	87	NT	100	83	86	100	60	100	57	NT	71	50	100	NT
Escherichia coli	131 (36.6%)	25	88	100	95	94	88	93	41	39	34	100	55	26	98	56
*Klebsiella* sp.	62 (17.3%)	7	83	100	88	89	83	84	54	63	56	0	67	53	76	100
Proteus mirabilis	11 (3%)	33	100	NT	91	100	54	82	29	63	54	NT	50	100	0	NT
*Pseudomonas* sp.	36 (10%)	12	85	72	82	93	74	81	60	0	0	100	67	80	100	50
Proteus vulgaris	9 (2.5%)	11	100	NT	100	100	100	100	43	50	25	NT	25	50	0	NT
*Salmonella *Typhi	1 (0.2%)	0	100	NT	NT	0	100	0	100	0	0	NT	100	NT	NT	NT
Neisseria gonorrhoeae	1 (0.2%)	NT	NT	NT	NT	NT	NT	NT	0	0	NT	NT	NT	NT	NT	NT

## Discussion

Antibiotic resistance in bacteria is posing a big concern in the realm of healthcare. We aimed to look at the antibiotic susceptibility patterns of Gram-positive and Gram-negative bacterial isolates at a tertiary care hospital. Our findings give insight into the predominance of specific bacterial species, their sensitivity to particular antimicrobial drugs, and the formation of an antibiogram. Our research uncovered a diverse array of bacterial strains, including 13 distinct species of Gram-positive and Gram-negative organisms. This diversity is typical of hospital settings, where a wide range of bacterial species coexist, and emphasizes the importance of tailored antimicrobial stewardship programs.

Antibiotic susceptibility trends in Gram-negative bacteria

In our analysis of Gram-negative bacteria, we identified 262 isolates. Among the Gram-negative bacterial isolates, *E. coli *and *Klebsiella* species were the predominant findings. This finding aligns with a previous study, indicating the widespread prevalence of *E. coli *in clinical settings [11]. *E. coli *is known for its adaptability and ability to develop resistance mechanisms, making it a subject of particular concern in healthcare-associated infections [12]. In a study conducted by Ghadiri et al. at Besat Hospital affiliated with Artesh University of Medical Sciences, they found that *E. coli *and *Klebsiella *bacteria were in line with their recognition as common pathogens in hospital-acquired infections, particularly UTI [13]. In our investigation, we observed a notably higher prevalence of *E. coli *bacteria in females (73.4%) compared to males. Our study aligns with known factors such as anatomical differences, hormonal influences, and behavioral practices that predispose women to UTI. This observation is consistent with prior research findings that have consistently reported a higher incidence of *E. coli *in female UTI patients compared to their male counterparts [14,15]. In our study, we observed notable patterns in the sensitivity profiles of *E. coli*. Specifically, we found that this bacterium exhibited considerable resistance to ampicillin, ceftriaxone, cefuroxime, and levofloxacin. However, we also noted a pronounced susceptibility to aztreonam, clindamycin, nitrofurantoin, and carbapenems. This underscores the importance of thoughtful antibiotic selection, particularly in the context of Gram-negative bacterial infections, as they are more likely to develop resistance to multiple antimicrobials [16]. Our analysis revealed that the majority of Gram-negative bacteria exhibited a notable susceptibility to carbapenem antibiotics, piperacillin/tazobactam, and amikacin. These findings align with the results of prior studies in this field, corroborating the consistency of these antibiotic sensitivity patterns [17,18].

Antibiotic susceptibility trends in Gram-positive bacteria

Among the Gram-positive bacteria under examination, we identified four discrete species: *Enterococcus* species, *S. aureus*, *Staphylococcus *coagulase-negative, and *Streptococcus *species. Notably, *S. aureus *emerged as the predominant isolate. *S. aureus *is a widely recognized human pathogen associated with a spectrum of infections, including those affecting the skin and soft tissues, pneumonia, and bloodstream infections [19]. In our analysis of sensitivity profiles among Gram-positive bacteria, we observed notable levels of susceptibility to chloramphenicol (99%), linezolid (98%), and ampicillin (95%). These findings align closely with those of a retrospective study conducted within a specialized healthcare setting in Saudi Arabia [20]. Their investigation, encompassing the susceptibility profiling of 10,870 Gram-positive isolates, also revealed substantial sensitivity toward linezolid (91.8%). However, in contrast to our results, their study indicated a more moderate sensitivity toward ampicillin (52.6%). In our study, *S. aureus *exhibited a notable susceptibility to several antimicrobials, including ampicillin, amoxiclav, cefazolin, cefotaxime, nitrofurantoin, teicoplanin, linezolid, and rifampin, all demonstrating 100% sensitivity. Conversely, higher resistance was observed for cotrimoxazole and vancomycin. Our findings align with a previous study, which similarly highlighted the susceptibility of *S. aureus* to linezolid, teicoplanin, and nitrofurantoin, along with its resistance to cotrimoxazole [21]. This resistance may be attributed to mechanisms such as permeability barriers, efflux pumps, mutations in target enzymes, or the acquisition of resistance by drug-resistant target enzymes in the case of cotrimoxazole [22]. Based on our study’s results, a significant proportion of Gram-positive bacteria have demonstrated a great susceptibility to beta-lactam antibiotics such as ampicillin, amoxiclav, ceftriaxone, and cefazolin. These findings align with prior research conducted in this field [23].

Cellular mechanisms of antimicrobial resistance in bacteria

Antimicrobial resistance in bacteria encompasses diverse cellular mechanisms that enable their survival in the face of antibiotic exposure. One fundamental strategy involves the understanding of drug targets, wherein bacteria alter the structure or function of the molecules that antibiotics bind to [24]. This can include the production of beta-lactamase enzymes that inactivate antibiotics like penicillin or the modification of penicillin-binding proteins to reduce antibiotic binding. Additionally, bacteria may reduce their cell membrane’s permeability, restricting the entry of antibiotics into the cell and effectively diminishing the drug’s effectiveness [25]. Moreover, the overexpression of efflux pumps, specialized transport proteins, allows bacteria to actively expel antibiotics from within their cells, lowering intracellular drug concentrations and conferring resistance [26]. Another layer of resistance mechanisms includes the production of target protective proteins that shield drug targets from antibiotic binding [27].

Bacteria have the ability to initiate self-repair mechanisms in response to damage caused by antibiotics. This process involves the MarA system, a regulatory mechanism within bacteria that is part of the Mar regulon, a set of genes regulated by MarA. The MarA system is often linked to inherent antibiotic resistance because it triggers the activation of genes essential for DNA repair and lipid transport [28]. Furthermore, a key function of the MarA system is to oversee the expression of efflux pumps that actively remove antibiotics from bacterial cells, reducing the amount of antibiotics inside the cells. This reduces the effectiveness of the antibiotics in combating the bacteria [29].

**Figure 2:**
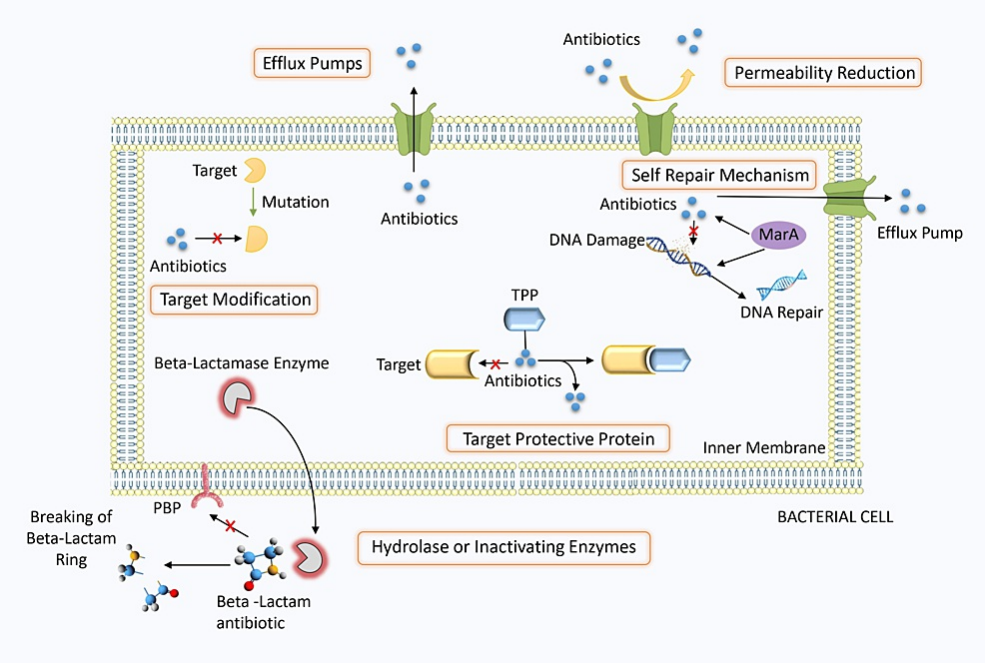
Mechanisms of antimicrobial resistance in bacteria PBP, penicillin-binding protein; TPP, target protective protein Image credit: Ashik Anil

Some bacteria produce hydrolases and inactivating enzymes that chemically modify antibiotics. These enzymes can cleave or modify the antibiotic molecule, rendering it inactive. For example, beta-lactamase enzymes can hydrolyze the beta-lactam ring in antibiotics like penicillin, leading to the loss of their antibacterial activity [30]. These multifaceted mechanisms collectively demonstrate the remarkable adaptability of bacteria to antimicrobial agents and emphasize the need for comprehensive strategies to combat drug-resistant bacterial infections. Understanding these mechanisms is paramount in the ongoing battle against antibiotic resistance. The mechanism behind the antimicrobial resistance in bacteria is illustrated and presented in Figure 2.

Limitations and future directions

In our study on analyzing antimicrobial sensitivity, we examined 357 bacterial isolates. However, it is crucial to recognize that our sample size, while substantial, might be relatively small when compared to broader epidemiological studies. This limitation could affect how broadly our findings can be applied to larger populations. Looking ahead, to enhance the strength of our insights, it is imperative to consider enlarging the sample size and conducting studies across multiple healthcare centers, which would offer a more robust assessment of antimicrobial sensitivity patterns. Additionally, given the increasing prevalence of resistance trends like extended-spectrum beta-lactamases and methicillin-resistant *S. aureus*, it is vital for future research to look into these resistance trends at a genetic and molecular level. This deeper understanding of the genetic basis of resistance not only paves the way for more precise treatment strategies but also contributes to global efforts to combat antimicrobial resistance effectively.

## Conclusions

Our research on antibiotic susceptibility profiling of both Gram-positive and Gram-negative bacterial isolates in a tertiary care hospital has provided valuable insights into the diversity of bacterial strains and their antimicrobial sensitivity patterns. With 357 isolates representing 13 distinct species, our study underscores the importance of a comprehensive antibiogram for informed clinical decision-making. Notably, the high prevalence of resistance mechanisms in Gram-negative bacteria, including *E. coli *and *Klebsiella *species, emphasizes the pressing need for effective strategies to combat multidrug resistance. Future research should prioritize expanding sample sizes, conducting multicenter studies, and exploring the genetic mechanisms behind resistance. These efforts will refine treatment strategies and strengthen the global fight against antimicrobial resistance, ultimately improving patient care and public health.
